# 急性髓系白血病首疗程疗效预测模型的构建和评价

**DOI:** 10.3760/cma.j.cn121090-20240816-00305

**Published:** 2025-04

**Authors:** 枫 竺, 一乐 周, 仪 张, 莉萍 毛, 德 周, 丽亚 马, 春梅 杨, 文娟 俞, 杏浓 叶, 菊英 韦, 海涛 孟, 敏 杨, 文渊 麦, 劼靖 钱, 艳玲 任, 引军 娄, 健 黄, 改香 许, 万灼 谢, 红艳 佟, 华锋 王, 洁 金

**Affiliations:** 1 浙江大学医学院附属第一医院血液科、浙江省血液肿瘤重点实验室、浙江省血液病临床医学研究中心，杭州 310000 Department of Hematology, First Affiliated Hospital, Zhejiang University, School of Medicine, Zhejiang Provincial Key Laboratory of Hematopoietic Malignancy, Zhejiang Provincial Clinical Research Center for Hematological Disorders, Hangzhou 310000, China; 2 舟山医院，舟山 316000 Zhoushan Hospital, Zhoushan 316000, China; 3 浙江大学癌症研究院，杭州 310000 Zhejiang University Cancer Center, Hangzhou 310000, China

**Keywords:** 白血病，髓系，急性, 首疗程疗效, 预测模型, Leukemia, myeloid, acute, Efficacy of the first course, Predictive model

## Abstract

**目的:**

明确急性髓系白血病（AML）首疗程缓解的相关因素，建立预测模型并对其进行评价。

**方法:**

收集2019年1月1日至2023年4月30日浙江大学医学院附属第一医院血液科收治的749例初诊AML患者临床资料，随机将其分为训练集和验证集，采用多因素Logistic回归模型分析与首疗程诱导缓解相关的变量，并建立相关预测模型。通过绘制预测模型的受试者工作特征曲线并计算曲线下面积，评估模型。

**结果:**

在训练集中分析与首疗程诱导缓解相关的独立因素为发病时外周血白细胞计数、CBFB::MYH11融合基因、CEBPA bZIP区突变、骨髓增生异常综合征（MDS）相关的基因突变，以及诱导化疗方案的选择。在上述基础上构建首疗程疗效预测模型，该模型在训练集和验证集曲线下面积为0.738（95％*CI*：0.696～0.780）和0.726（95％*CI*：0.650～0.801），Hosmer-Lemeshow检验结果显示*P*值分别为0.993和0.335。

**结论:**

本研究构建的模型对AML患者首疗程疗效具有良好的预测作用，为临床医师对AML患者预后判断及治疗方案的选择提供参考依据。

## Abstract (en)

**Objective:**

To identify the relevant factors for the first-course remission of acute myeloid leukemia（AML）and to develop a predictive model as well as assess its predictive capability.

**Methods:**

Clinical data of 749 patients newly diagnosed with AML admitted to the Department of Hematology, the First Affiliated Hospital, Zhejiang University, School of Medicine from January 1, 2019, to April 30, 2023, were collected and randomly divided into training and validation sets. Multivariate logistic regression analysis was conducted to determine variables associated with complete remission in the first course of induction therapy, and a predictive model was established based on these variables. The receiver operating characteristic（ROC）curve of the predictive model was plotted, and the area under the curve（AUC）was calculated.

**Results:**

The indicators predicting the first remission course included peripheral blood white blood cell count during onset, CBF::MYH11 fusion gene, CEBPA bZIP region mutation, myelodysplastic syndrome-related gene mutation, and induction chemotherapy regimen selection as independent factors for the first remission course. The model's area under the training and validation curves was 0.738（95% *CI*: 0.696–0.780）and 0.726（95% *CI*: 0.650–0.801）, respectively. The Hosmer-Lemeshow test results yielded *P*-values of 0.993 and 0.335, respectively.

**Conclusion:**

In this study, the developed model demonstrates a strong predictive capability for the efficacy of the first course of patients with AML, providing valuable guidance to clinicians in assessing patient prognosis and selecting appropriate treatment strategies.

急性髓系白血病（AML）是一组具有侵袭性、异质性的恶性疾病，以造血干细胞分化障碍和异常增殖为特征。目前首选的治疗是诱导化疗缓解后给予巩固化疗或造血干细胞移植以达到长期生存的目的。尽管近10年来靶向治疗及免疫治疗等治疗手段增多，但目前国际上各个中心报道的成人AML长期生存并没有明显改善[1]–[4]。我们之前的研究显示首疗程缓解和长期生存密切相关，首疗程获得完全缓解（CR）的AML患者以移植为节点的生存时间较首疗程未获得CR的患者显著延长[5]–[6]。为了探索首疗程缓解的相关因素并更好地指导临床治疗，我们回顾性分析了2019年至2023年在本中心治疗的新诊断的AML患者的临床资料，探讨首疗程缓解的相关因素、构建预测模型，并评价其有效性。

## 病例与方法

一、患者

回顾性收集2019年1月1日至2023年4月30日浙江大学医学院附属第一医院收治的749例初诊AML患者临床资料，包括基本信息（性别、年龄、初诊血常规、骨髓常规、融合基因、染色体、基因突变等）、治疗方案以及化疗后评价指标。排除标准：①急性早幼粒细胞白血病；②继发性AML转化前曾接受化疗；③首疗程评估前死亡；④患者缺初诊临床资料，如血常规、骨髓常规、融合基因、基因突变结果。所有患者的诊断均符合文献[7]标准。

二、定义

复合完全缓解（CRc）包括CR和伴血液学未完全恢复的CR（CRi）。CR定义为骨髓原始细胞<5％，外周血无原始细胞出现，髓外无白血病侵犯病灶，中性粒细胞计数≥1×10^9^/L且PLT≥100×10^9^/L。CRi定义为满足所有CR标准，但中性粒细胞计数<1×10^9^/L或PLT<100×10^9^/L。未缓解（NR）定义为患者目前评估的反应未达到CR、CRi的任何标准。微小残留病（MRD）阴性：流式细胞术检测MRD水平<0.1％，若获取细胞数低于100 000个，不纳入结果分析。

三、治疗方案

诱导方案包括以下几种：①强化疗方案：标准剂量的阿糖胞苷联合蒽环类药物，如DA/IA（阿糖胞苷100 mg/m^2^第1～7天联合去甲氧柔红霉素12 mg·m^−2^·d^−1^第1～3天或柔红霉素60 mg·m^−2^·d^−1^第1～3天）、HAA（高三尖杉酯碱2 mg·m^−2^·d^−1^第1～7天，阿克拉霉素20 mg/d第1～7天，阿糖胞苷100 mg/m^2^第1～7天）。②非强化疗方案：如CAG（阿糖胞苷10 mg/m^2^每12 h 1次，第1～14天；阿柔比星20 mg/d第1～4天；重组G-CSF 150 µg/m^2^每12 h 1次，第0～14天）、VA（维奈克拉100 mg/d 第1天，200 mg/d第3天，300 mg/d第4～21天；阿扎胞苷75 mg·m^−2^·d^−1^第1～7天）或VAA方案［维奈克拉100 mg/d第1天，200 mg/d第2天，400 mg/d第3～7天（根据评估骨髓增生情况延至第10天）；阿糖胞苷10 mg/m^2^每12 h 1次，第1～10天；阿扎胞苷75 mg·m^−2^·d^−1^第1～7天］。③新型方案：DAV（柔红霉素60 mg·m^−2^·d^−1^第1～3天；阿糖胞苷100 mg/m^2^第1～7天；维奈克拉100 mg/d第4天，200 mg/d第5天，400 mg/d第6～11天）或VHAA（高三尖杉酯碱2 mg·m^−2^·d^−1^第1～5天；阿糖胞苷100·m^−2^·d^−1^每12 h 1次，第1～5天；阿克拉霉素12 mg·m^−2^·d^−1^第1～5天；维奈克拉100 mg/d第2天，200 mg/d第3天，300 mg/d第4～10天）。

四、统计学处理

在训练集中，计量资料以中位数（*IQR*）表示，组间比较采用Mann-Whitney *U*检验；计数资料以频数（构成比）表示，组间比较采用卡方检验。以首疗程是否达到CRc为因变量，筛选变量为自变量，单因素Logistic回归分析模型中*P*值小于0.2的因素纳入多因素Logistic回归模型对变量进行筛选，确定最终有统计学意义的预测因素。通过绘制预测模型的受试者工作特性（ROC）曲线，计算训练集和验证集曲线下面积（AUC）及其95％*CI*，评价模型的区分能力。通过绘制预测概率与实际概率的校准曲线，采用Hosmer-Lemeshow检验，评价模型预测能力的一致性。在多因素Logistic回归分析结果的基础上，绘制预测模型列线图。组间比较和单因素、多因素Logistic回归采用SPSS26软件完成，双侧*P*<0.05为差异有统计学意义。ROC曲线、Hosmer-Lemeshow检验、列线图采用Ｒ统计软件4.0.2完成。

## 结果

一、患者基本情况

本研究共纳入749例患者，其中男380例，女369例，中位年龄55（14～93）岁，经1个疗程诱导治疗后达到CR 402例，CRi 79例，首疗程CRc率为64.2％，可纳入MRD评估患者的MRD转阴率为50.5％（350/693）。

二、训练集和验证集患者临床特征比较

按照3∶1比例随机将749例患者分割为训练集和验证集，训练集561例用于建立AML首疗程疗效预测模型，验证集188例用于验证预测模型。其中训练集中RUNX1::RUNX1T1阳性59例（10.5％），CBF::MYH11阳性42例（7.5％），TP53突变（突变频率大于10％）25例（4.5％），MDS相关基因突变阳性（定义为伴有≥２个下列基因突变：SRSF2、ZRSR2、SF3B1、ASXL1、BCOR、EZH2、U2AF1、STAG2、RUNX1）45例（8.0％）。根据MDS相关基因突变定义将患者分为三组：MDS相关基因突变阳性组、上述9个基因均阴性和仅有1个阳性组，三组CRc率分别为44.4％（20/45）、66.3％（262/395）和67.8％（82/121），差异有统计学意义（*P*＝0.011）。根据欧洲白血病网（ELN）2022分层，训练集中预后良好229例（40.8％），预后中等143例（25.5％），预后不良189例（33.7％）；训练集总的CRc率为65.1％（365/561），CRc_MRD-_率为51.9％（269/518）；其中112例（20.0％）患者给予新型方案治疗，CRc率为83.0％（93/112），CRc_MRD-_率为76.4％（81/106）。

验证集中RUNX1::RUNX1T1阳性19例（10.1％），CBF::MYH11阳性12例（6.4％），TP53突变（突变频率大于10％）12例（6.4％），MDS相关基因突变阳性16例（8.5％），其CRc率为50.0％（8/16），MDS相关基因突变均阴性和仅有1个阳性组CRc率分别为63.4％（83/131）和61.0％（25/41），三组比较CRc率差异无统计学意义（*P*＝0.655）。根据ELN2022分层，验证集中预后良好65例（34.6％），预后中等59例（31.4％），预后不佳64例（34.0％）；验证集总的CRc率为61.7％（116/188）,CRc_MRD-_率为46.3％（81/175）；其中33例（17.5％）患者给予新型方案治疗，CRc率为93.9％（31/33），CRc_MRD-_率为84.4％（27/32）。

两组患者的临床特征比较见表1，两组之间其他基线特征如发病年龄、性别、初诊WBC、初诊骨髓原始细胞比例、核型和预后分层、诱导方案选择等差异均无统计学意义，而两组患者间初诊HGB水平差异有统计学意义。

**表1:** 训练集和验证集临床资料的比较

变量	训练集（561例）	验证集（188例）	统计量	*P*值
发病年龄［岁，*M*（*IQR*）］	54（41.0～64.0）	57（43.0～66.8）	2.493	0.115
女性［例（％）］	272（48.4）	97（51.6）	0.545	0.257
外周血WBC［×10^9^/L，*M*（*IQR*）］	11.7（2.8～43.8）	13.2（2.8～33.1）	1.830	0.177
HGB［g/L，*M*（*IQR*）］	84.0（70.0～104.0）	80.0（64.0～99.8）	4.810	0.029
PLT［×10^9^/L，*M*（*IQR*）］	52.0（27.0～97.8）	44.0（27.0～80.0）	3.290	0.070
骨髓原始细胞［％，*M*（*IQR*）］	62.0（43.0～78.0）	61.0（42.0～80.0）	0.011	0.917
染色体分层［例（％）］				
良好	56（10.0）	14（7.4）	1.609	0.447
中等	428（76.3）	145（77.1）		
不良	51（9.1）	21（11.2）		
未知^a^	26（4.6）	8（4.3）		
融合基因［例（％）］				
RUNX1::RUNX1T1	59（10.5）	19（10.1）	0.025	0.873
KMT2A重排	48（8.6）	14（7.4）	0.228	0.633
CBF::MYH11	42（7.5）	12（6.4）	0.256	0.745
基因突变［例（％）］				
DNMT3A	121（21.6）	45（23.9）	0.458	0.543
NPM1	124（22.1）	33（17.6）	1.760	0.185
FLT3-ITD^b^	101（18.0）	43（23.2）	2.452	0.117
CEBPA bZIP	68（12.1）	23（12.2）	0.002	0.967
MDS相关基因突变^c^	45（8.0）	16（8.5）	0.045	0.832
TP53^d^	25（4.5）	12（6.4）	1.102	0.294
ELN2022分层［例（％）］				
低危	229（40.8）	65（34.6）	3.218	0.200
中危	143（25.5）	59（31.4）		
高危	189（33.7）	64（34.0）		
首疗程方案［例（％）］				
强化疗方案	242（43.1）	72（38.3）	3.121	0.210
非强化疗方案	207（36.9）	83（44.1）		
新型方案	112（20.0）	33（17.6）		
疗效［例（％）］				
CRc	365（65.1）	116（61.7）	0.692	0.406
CRc_MRD-_^e^	269（51.9）	81（46.3）	1.667	0.197

**注** MDS：骨髓增生异常综合征；ELN：欧洲白血病网；CRc：复合完全缓解；MRD：微小残留病。^a^34例患者缺初诊时骨髓染色体结果；^b^有4例患者因初诊无基因突变原始结果，无法明确是否合并FLT3-ITD突变；^c^MDS相关基因突变指≥2个下列基因突变阳性：SRSF2、ZRSR2、SF3B1、ASXL1、BCOR、EZH2、U2AF1、STAG2、RUNX1；^d^TP53突变阳性指TP53基因突变量超过10％；^e^训练集和验证集分别有518例和175例可评估MRD

三、首疗程疗效预测模型的构建

在训练集中应用单因素和多因素分析结果显示，5个变量和首疗程化疗疗效相关（表2），其中伴有CBFB::MYH11融合基因阳性或CEBPA bZIP突变以及应用新型治疗方案诱导治疗的首疗程疗效更好；而发病时高WBC、MDS相关基因突变阳性者首疗程疗效较差。根据这5个变量加患者年龄、发病时骨髓原始细胞、TP53基因突变绘制列线图（图1），构建了首疗程疗效预测模型用于预测AML患者首疗程获得CRc的概率，并以此开发了终端网络在线计算器（https://zju-aml.shinyapps.io/AML-CRc/）。

**表2:** 训练集中和首疗程缓解相关的单因素及多因素Logistic回归分析

变量	单因素			多因素		
	*OR*值	95％*CI*	*P*值	*OR*值	95％*CI*	*P*值
年龄	0.551	0.373～0.815	0.004	0.677	0.426～1.076	0.099
性别	1.149	0.812～1.627	0.433			
外周血WBC	0.993	0.990～0.997	<0.001	0.993	0.988～0.997	0.001
外周血HGB	1.009	1.001～1.017	0.020	1.007	0.998～1.016	0.106
外周血PLT	0.997	0.995～1.000	0.024	0.998	0.995～1.000	0.102
骨髓原始细胞计数	0.990	0.982～0.998	0.016	0.991	0.981～1.000	0.053
分层						
染色体分层	0.612	0.408～0.920	0.018	1.069	0.564～2.025	0.839
ELN2022分层	0.577	0.468～0.711	<0.001	0.924	0.674～1.267	0.626
融合基因						
CBF::MYH11	11.938	2.854～49.948	0.001	10.651	2.332～48.655	0.002
RUNX1::RUNX1T1	1.654	0.895～3.055	0.108	1.297	0.521～3.226	0.576
KMT2A重排	0.977	0.526～1.814	0.942			
基因突变						
CEBPA bZIP	3.514	1.753～7.044	0.001	3.869	1.712～8.743	0.001
MDS相关基因突变^a^	0.397	0.214～0.734	0.003	0.429	0.204～0.903	0.026
TP53^b^	0.402	0.179～0.903	0.027	0.430	0.162～1.141	0.090
FLT3-ITD	0.550	0.356～0.852	0.007	0.834	0.484～1.438	0.514
DNMT3A	0.566	0.376～0.853	0.006	1.122	0.681～1.849	0.652
NPM1	0.811	0.537～1.224	0.319			
化疗方案	3.185	1.878～5.403	<0.001	3.141	1.766～5.587	<0.001

**注** ELN：欧洲白血病网。^a^骨髓增生异常综合征（MDS）相关基因突变指≥2个下列基因突变阳性：SRSF2、ZRSR2、SF3B1、ASXL1、BCOR、EZH2、U2AF1、STAG2、RUNX1；^b^TP53突变阳性指TP53基因突变量超过10％

**图1:**
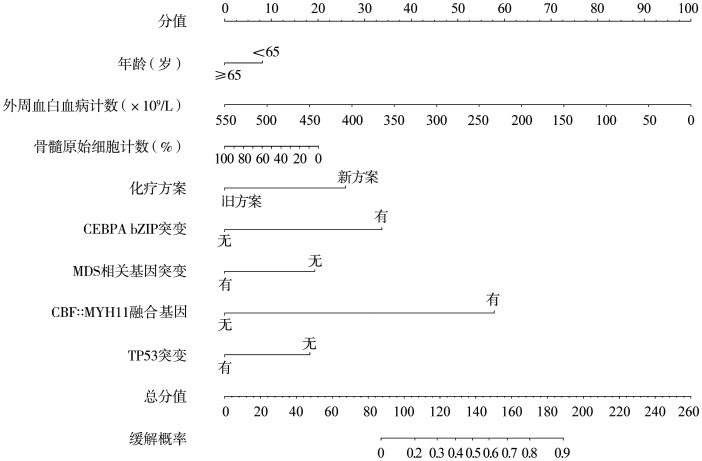
急性髓系白血病首疗程疗效预测模型列线图 **注** 化疗方案：维奈克拉联合强化疗定义为新方案，其他均定义为旧方案。骨髓增生异常综合征（MDS）相关基因突变：≥2个下列基因突变阳性定义为有突变：SRSF2、ZRSR2、SF3B1、ASXL1、BCOR、EZH2、U2AF1、STAG2、RUNX1；阴性或单个阳性定义为无突变

四、预测模型的区分能力和一致性评价

在多因素回归模型的基础上，计算训练集每个个体的预测概率，并绘制ROC曲线，计算ROC曲线的AUC及其95％*CI*，评价预测模型的区分能力。结果显示，训练集预测模型ROC曲线的AUC为0.738（95％*CI*：0.696～0.780）；用同样的方法绘制验证集人群ROC曲线，计算AUC及其95％*CI*，评价预测模型在验证集人群中的区分能力。结果显示，验证集预测模型ROC曲线的AUC为0.726（95％*CI*：0.650～0.801），提示预测模型在验证集人群中仍具有较高的区分能力（图2）。进一步绘制预测概率与实际概率的校正曲线，对模型的预测能力进行一致性评价。结果显示，校正曲线基本分布在图形对角线位置，预测概率与实际概率之间基本吻合，Hosmer-Lemeshow检验结果显示训练集*P*值0.993，验证集*P*值为0.335，提示模型预测能力的一致性好。

**图2:**
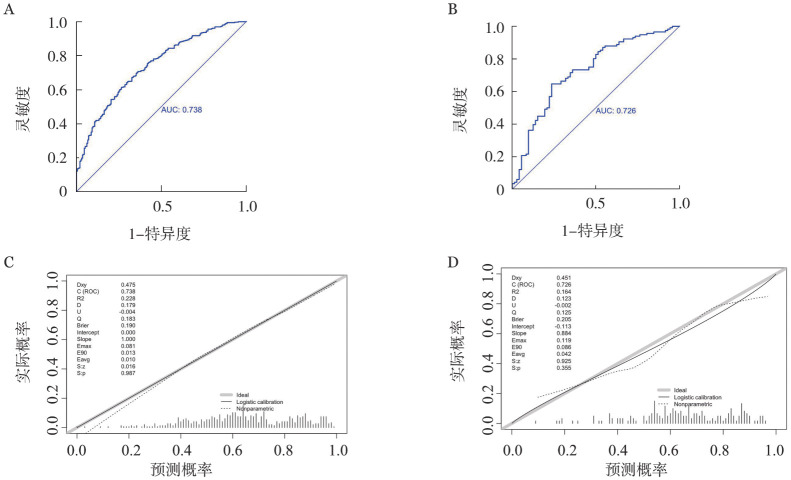
预测模型的区分能力和一致性评价 **A** 训练集ROC曲线；**B** 验证集ROC曲线；**C** 训练集校准曲线；**D** 验证集校准曲线

五、以CRc _MRD-_为结局的预测模型区分能力和一致性评价

我们对于可评估MRD的693例患者以CRc_MRD-_为结局进行预后因素的统计分析，单因素结果显示发病年龄、初诊外周血WBC、HGB、PLT、ELN2022分层、CBF::MYH11、CEBPA bZIP、MDS相关基因突变、TP53突变、DNMTA、化疗方案选择和预后相关，将单因素结果中*P*值小于0.2的因素纳入多因素分析，结果显示，ELN2022分层、初诊外周血WBC、CBF::MYH11、CEBPA bZIP、TP53突变、化疗方案选择是独立预后因素，基于上述7个变量建立预测模型，训练集和验证集ROC曲线的AUC分别为0.643（95％*CI*：0.595～0.690）和0.574（95％*CI*：0.490～0.663），Hosmer-Lemeshow检验结果显示训练集和验证集*P*值均小于0.001，说明预测值与真实值之间有非常明显的差异，未来可能需要更大量的数据来建立以CRc _MRD-_为结局的预测模型。

## 讨论

AML是一种常见的恶性血液病，虽然近几年来新药不断被开发，但AML患者的长期生存没有明显改善[1]–[4]，目前根据ELN2022指南定义2个标准方案诱导化疗未缓解为难治性AML[8]，我们之前的研究表明首疗程达到缓解有生存获益，但目前尚无大型研究系统性说明首疗程缓解的相关因素，亦无疗效预测模型的建立。

我们在研究中发现分子生物学改变和首疗程疗效密切相关。在ELN2022指南中，具有CEBPA bZIP突变患者为预后良好，Taube等[9]通过回顾性分析4 708例AML患者资料显示，存在bZIP区域内突变患者，无论是单位点突变和双位点突变，生存均优于无bZIP区域内突变的患者。本研究证实是否存在CEBPA bZIP区域内突变是首疗程缓解的独立影响因素。Lindsley等[10]通过分析433例AML患者的二代测序结果，发现具有SRSF2、ZRSR2、SF3B1、ASXL1、BCOR、EZH2、U2AF1及STAG2基因突变患者诊断继发性AML有高度特异性，这些患者具有缓解率低、生存时间短的预后特征，且被认为与原发AML有着不同的发病机制，在ELN2022指南中也将具有包括上述8个基因及RUNX1基因突变定义为预后不良，本研究比较上述基因全阴性、1个阳性、2个及以上阳性患者首疗程疗效，后者与前两者缓解率差异有统计学意义，因此将两个及以上上述基因突变阳性视作具有MDS相关基因突变，多因素分析证实其为首疗程不缓解的独立预后因素。CBFB::MYH11融合基因阳性的这部分AML患者属于预后良好组，缓解率高达88％，达沙替尼或强烈方案能提高缓解率和长期生存[11]–[13]，本研究中CBF::MYH11融合基因为独立预后因素，和文献报道一致。以往报道FLT3-ITD患者预后较差，在ELN2017指南中高频FLT3-ITD归于预后不良组，ELN2022指南更新为无论突变频率，均归至中危组，在本研究中FLT3-ITD与首疗程疗效无明显相关，其原因有可能为FLT3-ITD突变预后不良主要体现在高复发率[14]–[15]。

DA方案作为AML的标准诱导方案已有40余年，但仅有60％～80％患者能通过DA方案获得缓解，且5年生存低于50％[1]–[2],[4],[16]，本中心提出的HAA（高三尖杉酯碱+阿克拉霉素+阿糖胞苷）方案，总缓解率达到73％[3]，但老年或体弱AML患者难以耐受上述强烈方案。维奈克拉是一种高选择性bcl-2抑制剂，维奈克拉联合小剂量阿糖胞苷或去甲基化药物等方案能明显提高75岁以上或者体弱患者诱导缓解率并改善长期生存。本中心提出的DAV方案（DA方案联合维奈克拉）诱导缓解率达91％[5]，远高于以往的临床研究结果。本研究预测模型结果显示强化疗方案联合维奈克拉有利于诱导缓解，与以往研究结果一致[5],[16]–[19]。

通过预测模型能早期识别首疗程不缓解的高危因素，且进一步通过列线图或在线计算器，在预测模型中输入初诊患者的特征值，从而预测其应用不同治疗方案的疗效，给医师选择最优方案提供参考。但本研究为单中心回顾性研究，无外部验证数据，可能存在一定选择偏倚。

综上，AML首疗程诱导治疗疗效可能与发病时外周血WBC、CBF::MYH11融合基因、CEBPA bZIP基因突变、具有MDS相关的基因突变、诱导化疗方案相关。本研究构建的临床预测模型可以较好地预测不同诱导方案对于不同特征初诊患者的首疗程缓解率，有助于临床医师治疗方式的选择，但还需外部验证来确定其临床有效性。
